# Determination of the differentially expressed genes in microarray experiments using local FDR

**DOI:** 10.1186/1471-2105-5-125

**Published:** 2004-09-06

**Authors:** J Aubert, A Bar-Hen, J-J Daudin, S Robin

**Affiliations:** 1 UMR INAPG/INRA/ENGREF 518, 16, rue C. Bernard, 75231 Paris Cedex 05, France

## Abstract

**Background:**

Thousands of genes in a genomewide data set are tested against some null hypothesis, for detecting differentially expressed genes in microarray experiments. The expected proportion of false positive genes in a set of genes, called the False Discovery Rate (FDR), has been proposed to measure the statistical significance of this set. Various procedures exist for controlling the FDR. However the threshold (generally 5%) is arbitrary and a specific measure associated with each gene would be worthwhile.

**Results:**

Using process intensity estimation methods, we define and give estimates of the local FDR, which may be considered as the probability for a gene to be a false positive. After a global assessment rule controlling the false positive error, the local FDR is a valuable guideline for deciding wether a gene is differentially expressed. The interest of the method is illustrated on three well known data sets. A R routine for computing local FDR estimates from *p*-values is available at .

**Conclusions:**

The local FDR associated with each gene measures the probability that it is a false positive. It gives the opportunity to compute the FDR of any given group of clones (of the same gene) or genes pertaining to the same regulation network or the same chromosomic region.

## Background

Microarrays are part of a new class of biotechnologies that allow the monitoring of the expression level of thousands of genes simultaneously. Among the applications of microarrays, an important task is the identification of differentially expressed genes, i.e genes whose expressions are associated with the status of the patient (treatment/control for example).

The biological question of the identification of differentially expressed genes can be restated as a one (for paired data) or two-sample (for unpaired data) hypothesis testing procedure: is the gene differentially expressed between the two situations? However, when thousands of genes in a microarray data set are evaluated simultaneously by fold changes or significance tests approach, multiple testing problems immediately arise and lead to many false positive genes. In this 'one-by-one gene' approach the probability of detecting false positives rises sharply.

The False Discovery Rate (FDR), is defined as the expected fraction of false rejections among those hypotheses rejected. In their seminal paper Benjamini & Hochberg [1] provided a distribution free procedure (BH) for choosing a threshold on *p*-values that guarantees that the FDR is less than a target level α. The same paper demonstrated that the BH procedure is more powerful than the Bonferroni method that controls the familywise error rate.

The FDR gives an idea of the expected number of false positive hypotheses that a practitioner can expect if the experiment is done an infinite number of time. As usual with expectation, it gives very little information about the number of false discovery hypotheses in a given experiment.

### Motivation

The value of 1, 5 or 10% for the FDR, which determines the threshold *t*, is arbitrary. Storey and Tibshirani [2] stressed the importance of assessing to each feature its own measure of significance. They proposed to use the *q*-value,


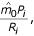


where *P*_*i *_is the *p*-value of the ordered gene *i*, *R*_*i *_is the total number of rejected genes whose *p*-values are less than the threshold *t *= *P*_*i *_and 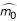 is an estimate of the total number of non differentially expressed genes, *m*_0_.

The *q*-value is appealing because it gives a measure of significance that can be attached to each gene, but it must be stressed that it is not an estimate of the probability for the gene to be a false positive. The *q*-value is generally lower than the latter because it is computed using all the genes that are more significant than gene *i*. Obviously a gene whose *p*-value is near to the threshold *t *does not have the same probability to be differentially expressed than a gene whose *p*-value is close to zero. Therefore the *q*-value gives a too optimistic view of the probability for the gene to be a false positive.

Therefore it is interesting to obtain an estimate of the FDR attached to each gene, called local FDR, from an inferential point of view and without any assumption about the distribution of the *p*-values under *H*_1_.

## Results

Let

*H*_0_(*i*) = {gene *i *is not differentially expressed}.

Let the local FDR be the probability that a given gene is not differentially expressed. More specifically, *FDR*(*i*) is the probability that a gene, whose *p*-value is *P*_*i*_, is not differentially expressed, taking into account the whole set of tests. A raw local FDR estimate is defined in a first step. In a second step the local FDR estimate is defined as a smoothed value based on the raw values.

Let *P*_1 _< … <*P*_*m *_denote the ordered *p*-values for testing *H*_0_(*i*). The raw local FDR estimate for gene *i *is:


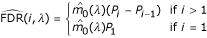


where


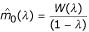


where λ is a tuning parameter and *W*(λ) = #{*i*, *P*_*i *_> λ}, see Storey [3].

Assume that the *p*-values for the non-differentially expressed genes are independent. The raw local FDR estimate has the following properties:

• Under *H*_0_(*i*) and *H*_0_(*i *- 1) and if *E*(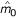) = *m*_0_, 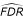
(*i*, λ) is unbiased with mean 1.

• Let 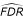
(*i*, *m*_0_) = *m*_0_(*P*_*i *_- *P*_*i*-1_). Under *H*_0_(*i*) and *H*_0_(*i *- 1) and if *m*_0 _is known, *V*(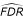
(*i*, *m*_0_)) = 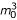/[(*m*_0 _+ 1)^2^(*m*_0 _+ 2)] ≈ 1, for *m*_0 _large enough. This value is a lower bound for *V*(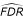
(*i*, λ)) when *m*_0 _is unknown.

• The variance of the raw local FDR under *H*_1 _is generally much smaller than under *H*_0_.

• 
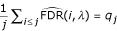
 where *q*_*j *_is the *q*-value of gene *j*. The *q*-value may thus be viewed as the mean of the local FDR of the genes with *p*-values lower than *P*_*j*_.


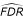
(*i*, λ) is generally a very variable estimator. Moreover the local FDR should increase with the *p*-value. This is not the case for the raw local FDR. Therefore it is necessary to use a smoothed estimate.

The smoothed local FDR(*i*) is


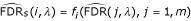


where *f*_*i *_is a smoothing function of the 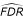
(*j*, λ) for *j *= 1, *m*, computed at position *P*_*i*_.


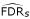
(*i*, λ) gives a very valuable guideline for the choice of a threshold. One may consider the curve of the local FDR versus the index of the gene ordered by their *p*-values: a good candidate for the threshold should be a point with a high second order derivative, which corresponds to an abrupt change in the slope of the curve (see the examples of the following section). The second order derivative of the smoothed local FDR can be computed numerically using finite differences.

As an interesting application of the local FDR, it is possible to compute the FDR associated with a class of genes or clones by summing up the local FDR estimate of each clone or gene: one may consider for example clones corresponding to the same gene, genes known involved in a given regulatory network, or gene from the same chromosomic region, and associate a FDR with the whole class. These genes do not need to have consecutive *p*-values. The following sections demonstrate how the local FDR can be useful using the data of well known experiments.

### Local FDR on Golub data set

Golub [4] were interested in identifying genes that are differentially expressed in patients with two types of leukemias (ALL, AML). Gene expression levels were measured using Affymetrix high-density chips containing 6817 human genes. The learning set comprises 27 ALL cases and 11 AML cases.

Data are available in the R multtest package. We used the preprocessing proposed by the authors and the *p*-values based on random permutations of the ALL/AML labels on Welch *t*-statistics for each gene, Dudoit [5], on the 3051 remaining genes. *m*_0 _is estimated with bootstrap method as suggested by Storey and Tibshirani and implemented in the library GeneTS of software R.

**Figure 1:**
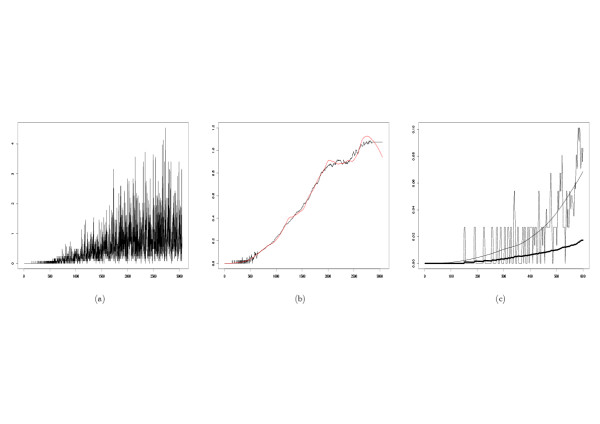
**Plots of the local FDR estimate for Golub data ***x*-axis: index of genes ordered along their *p*-values, *y*-axis: local FDR estimate. (a): raw values, (b): smooth estimates: moving average (discrete jumps), lowess (smooth curve), (c): zoom on the first 600 genes of (b): moving average (discrete jumps), lowess (upper smooth curve), *q*-value (lower thick smooth curve).

Figure 1(a) presents the 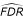
(*i*) for ordered genes and 1(b) presents the smooth curves obtained using lowess with a span of 0.2 and an adaptative moving average method.

We can see that there is an abrupt change of the smoothed local FDR around gene number 500 which corresponds to a threshold *t *= 0.15 for the *p*-value. This may be an indication about the threshold. The Figure 1(c) presents a zoom of the Figure 1(b) for the first 600 *p*-values. We can see in Figure 1(c) that if we select the 438 (14%) top genes, we obtain a *q*-value equal to 0.0078 while the 438^*th *^gene has a local FDR equal to 0.027. It must be noticed that there is a big difference between the two measures of FDR because the numerous regulated genes with very small *p*-values have a great influence on the *q*-value, which is not the case of the local FDR (see Figure 1(c)).

The *p*-values have been obtained using random permutations. Therefore the *p*-values are discrete with several genes possessing the same *p*-value. Therefore the values of 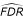
(*i*, λ) may be equal to 0 because the difference between two successive *p*-values is 0. The discrete structure of the *p*-values implies a departure from the theoretical continuous uniform distribution. This explains why the moving average smoothing creates discrete jumps which appear in Figure 1(c).

If the distribution of the statistics under *H*_0 _is correct, the *p*-values are distributed as a uniform distribution over [0, 1]. The empirical distribution of the high observed *p*-values (say above 0.5) is far from the uniform distribution. There are several non-exclusive possibilities to explain this: more than 50% of the genes are differentially expressed, the gene results for non-differentially expressed are correlated or there is a technical problem in the random permutations of the Welch *t*-statistics.

### Local FDR on Breast Cancer data set

Storey and Tibshirani [2], have analysed in detail data from Hedenfalk [6] on 15 microarrays on breast cancer. Using the same *p*-values, we have computed local FDR estimates. The three genes which have been analysed in detail by Storey and Tibshirani [2] are presented in Table 1.

One can see that the smooth local FDR estimate is generally greater than the *q*-value and gives a better idea of the probability that a gene is a false positive. For example, at the level of 5%, CTGF will be considered as differentially expressed on the basis of the *q*-value while it will be considered as non differentially expressed using the local FDR.

**Figure 2:**
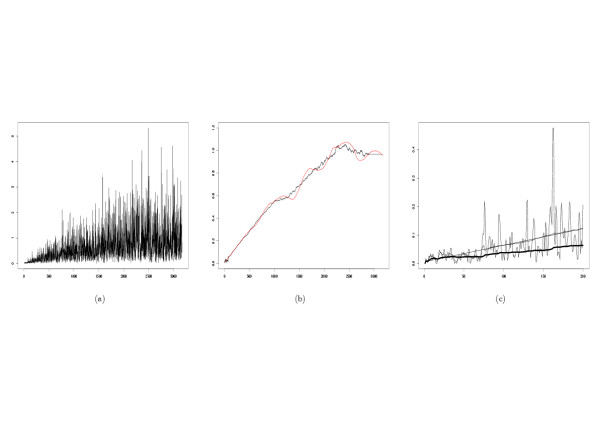
**Plots of the local FDR estimate for Hedenfalk data ***x*-axis: index of genes ordered along their *p*-values, *y*-axis: local FDR estimate. (a): raw values, (b): smooth estimates: moving average (discrete jumps), lowess (smooth curve), (c): zoom on the first 200 genes of (b): raw values (discrete jumps), moving average and lowess (smooth curves), *q*-value (lower thick smooth curve).

Figure 2(a) presents the 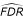
(*i*) for ordered genes and 2(b) presents the smooth curves obtained using lowess with a span of 0.2 and moving average methods. The two smoothing methods give similar results.

Setting λ = 0.5, Storey and Tibshirani [2] estimate that 67% of the 3170 genes in the data are not differentially expressed. The asymptote near 1 of the smooth curve supports this estimation.

### Local FDR on ApoAi data

**Figure 3:**
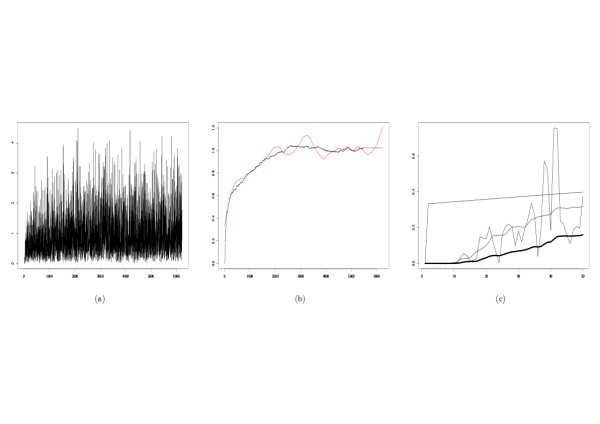
**Plots of the local FDR estimate for Apo-AI data ***x*-axis: index of clones ordered along their *p*-values, *y*-axis: local FDR estimate. (a): raw values, (b): smooth estimates: moving average (small discrete jumps), lowess (smooth curve), (c): zoom on the 50 first genes of (b): raw values (discrete jumps), moving average (smooth curve) lowess (upper rectangular curve), *q*-value (lower thick smooth curve).

The goal of the study is to identify genes with altered expression in the livers of two lines of mice with very low HDL cholesterol levels compared to inbred control mice. The mouse model is the apolipoprotein AI (ApoAI) knock-out mice. ApoAI is a gene known to play a pivotal role in HDL metabolism. The statistical analysis is described in Dudoit [7]. Height clones are expected to be differentially expressed between the control and the knock-out mices because they are clones of the ApoAI gene or of genes coregulated with ApoAI. The height clones are actually the 8 top clones detected by the statistical tests. However there are other following clones which seem statistically significant if we consider the *q*-value. We can see on the Figure 3(c) that the local FDR values are much higher than the *q*-values.

Figure 3(a) presents the 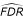
(*i*) for ordered clones and Figure 3(b) presents the smooth curves obtained using lowess with a span of 0.2 and moving average methods. The two smoothing methods give different results at the two ends of the [0, 1] interval. The moving average method which uses a special adaptative algorithm for the ends gives a better smoothing. This is particularly important for the clones with a small *p*-value for which it is crucial to obtain good estimates of the probability of being false positives. The lowess smoothing does not work well for the 50 first clones. In this particular case the default smoothing parameter *f *= 0.2 is not well suited and should be lower. However if it is chosen too low, the smoothing will not fit well the rest of the curve.

There are two clones of the gene Apo-AI. If we want to estimate the FDR of these two clones taken in a whole, we compute the mean of the smoothed local FDR of the two clones (the first and the height top clones) and obtain a local FDR for the gene Apo-AI, which is equal to 
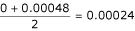
. This example shows that it is possible to estimate the local FDR of any group of clones. This opportunity provided by the local FDR is certainly one of its major advantage with many potential applications.

## Discussion

The curve of the smoothed local FDR is an efficient tool to summarize the information about the number and the statistical significance of differentially expressed genes, and may also be used to give an indication about the validity of the statistical assumptions. Moreover it is a valuable tool to choose the threshold for separating the differentially expressed genes from the non-differentially expressed one: one can choose a value of *t *maximizing the second derivative. Alternatively one can use a cost function and choose the threshold that minimizes the mean cost for a given cost function: using cost of the experiment, cost of false positive gene validation and the profit of discovering a differentially expressed gene, it is direct to compute the optimal strategy for choosing the threshold.

Note that a decision rule based on the local FDR would lead to a different set of selected genes than the usual one obtained by controlling the FDR. Consider the set of tests for which the local FDR is below 0.05, say. This set is not identical to the set identified by the standard criterion that *FDR *< 0.05. The local FDR is higher than the *q*-value. Therefore the first set is strictly included in the second one. The local FDR rule is therefore more conservative than the usual FDR one.

## Conclusions

The *p*-value gives the probability that a non differentially expressed gene would be as or more extreme than the gene under concern. The *q*-value indicates the estimated proportion of genes as or more extreme than the gene under concern that are a false positive. The local FDR gives the estimated proportion of genes around the gene under concern which are false positive. The latter may be used as the probability that the gene under concern is a false positive, taking into account the multiplicity of the test. One of the major interest of the local FDR is that it gives the opportunity to compute the FDR of any given group of clones (of the same gene) or genes pertaining to the same regulatory network or the same chromosome.

## Methods

### Model

Basically, the various procedures proposed in the literature aim to test the null hypothesis

*H*_0_(*i*) = {gene *i *is not differentially expressed}.

Let consider a particular experiment. We observed the differential expression of the genes and compute the associated ordered *p*-values *P*_*i*_. In the following we will use the classical property: the *p*-values corresponding to non differentially expressed genes are uniformly distributed over [0, 1]. Furthermore, we will assume, as often, that these *p*-values are independent. However, the independence of the *p*-values of differentially expressed genes is not required. Consider a multiple testing situation in which *m *tests are being performed. Let *m*_0 _be the number of non differentially expressed genes. Let *I*(*t*) be the set of the genes having a *p*-value lower than *t*: *I*(*t*) = {*i *: *P*_*i *_≤ *t*} and *R*(*t*) = #*I*(*t*), its cardinal. Let

*V*(*t*) = #[*I*(*t*) ∩ (*i *∈ *H*_0_)]

and

*S*(*t*) = #[*I*(*t*) ∩ (*i *∈ *H*_1_)].

Using a threshold *t*, the *m *genes can be classified according to the following 2 × 2 table 2:

The Family Wise Error Rate (FWER) is defined to be

FWER = *P *[*V*(*t*) ≥ 1].

A classical way to control FWER is given by the Bonferroni inequality. This quantity corresponds to the most direct extension from a test hypothesis procedure but can be very restrictive in a multiple testing procedure.

The status of the gene associated with the *P*_*i *_is an unobserved value. It is the same framework as point process (see for example [8]). In fact we observe *R*(*t*) = *V*(*t*) + *S*(*t*) the sum of two counting processes. The first one *V*(*t*) is a counting process associated with non differentially expressed gene. Since the *p*-values under *H*_0 _are uniformly distributed, *V*(*t*) has a binomial distribution with parameter *m*_0 _and *t*. The intensity of *V*(*t*) is constant and proportional to *m*_0_. *S*(*t*) is the counting process associated with gene under *H*_1 _and very few can be said about its distribution. One may expect the intensity of *S*(*t*) to be decreasing with *t*. The false discovery rate is defined as:


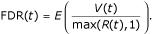


It corresponds to the expected proportion of rejections that are incorrect.

The BH procedure works as follows. Let *P*_1 _< … <*P*_*m *_denote the ordered *p*-values. Calculate *k *= max_*i*_{*P*_*i *_≤ α*i*/*m*}. The procedure rejects all null hypotheses for which *P*_*i *_≤ *P*_*k*_. If the tests are independent, this procedure ensures that


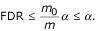


Let FDR(*t*) be the FDR when rejecting all null hypotheses with *P*_*i *_≤ *t*. Because the *p*-values of non-differentially expressed genes are uniformly distributed over [0, 1], a natural estimate of FDR(*t*) is


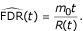


Therefore the problem is to estimate *m*_0_. Storey [3], proposed to estimate *m*_0 _with


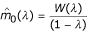


where λ is a tuning parameter. In particular the case λ = 0 leads to 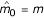. This is the most conservative case and corresponds to the BH procedure. Since the practical implementation of Storey method gives reasonably good results, we used it in the examples.

FDR is defined as the expectation of the ratio of two counting processes *V*(*t*) and *R*(*t*): FDR(*t*) = *E*[*V*(*t*)/max(*R*(*t*), 1)]. The expectation of *V*(*t*) is *m*_0_*t *and *R*(*t*) is observed. Therefore, Storey [3] propose to use the following estimate:


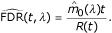


The ratio of the expectations differs from the expectation ratio but Storey [3] proved that *E*(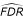
(*t*, λ)) ≥ FDR(*t*) using a convexity argument.

### Definition and Estimation of the Local FDR

As stated before, *V*(*t*) and *R*(*t*) are counting (i.e. cumulative) processes. It would be very interesting to estimate the ratio of the local intensities of the two processes at point *t*. The intensity of process *V*(*t*) is equal to *m*_0 _and thus is known, provided that we know *m*_0_. The intensity of process *R*(*t*) is unknown, but *R*(*t*) is observed. Therefore, using point process methods it is possible to estimate its intensity at each point *t*.

We first define the cumulative processes from *t*_1 _to *t*_2_:

Let 0 ≤ *t*_1 _<*t*_2_, *I*(*t*_1_, *t*_2_) = {*i *: *t*_1 _<*P*_*i *_≤ *t*_2_},

*R*(*t*_1_, *t*_2_) = #*I*(*t*_1_, *t*_2_),

*V*(*t*_1_, *t*_2_) = #[*I*(*t*_1_, *t*_2_) ∩ (*i *∈ *H*_0_)]

and

*S*(*t*_1_, *t*_2_) = #[*I*(*t*_1_, *t*_2_) ∩ (*i *∈ *H*_1_)].

FDR (*t*_1_, *t*_2_) is defined as the expected ratio of *V *(*t*_1_, *t*_2_) and *R*(*t*_1_, *t*_2_):


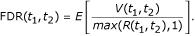


It is a generalization of the usual FDR: if *t*_1 _= 0 and *t*_2 _= *t *then FDR(*t*_1_, *t*_2_) = FDR(*t*). So, the natural estimate of FDR(*t*_1_, *t*_2_) is:


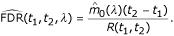


The substitution of 0 by *t*_1 _does not change the proof, so using the same convexity argument as Storey [3], we obtain the following property:

*E*(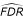
(*t*_1_, *t*_2_, λ)) ≥ FDR (*t*_1_, *t*_2_).

The local FDR is the FDR(*t*_1_, *t*_2_) for small intervals [*t*_1_, *t*_2_]. If we want to estimate the local FDR around the *p*-value of the gene *i*, the question can be restated as how to estimate the ratio of the intensities of two processes around a given point *P*_*i*_.

The intensity of process *R*(*t*) has to be estimated at each value of *t*. It is possible to consider small windows of size *h*, or alternatively, to consider windows of different sizes corresponding to a fixed count for *R*(*t*). We have chosen the latter solution, for windows of variable size seem more appealing in the particular context.

Let FDR(*i*) be the local FDR around *P*_*i*_. To estimate FDR(*i*) we need to define a neighborhood around *P*_*i*_. Let *V*_*i *_= *V*(*P*_*i*-1_, *P*_*i*_). Remarking that *R*(*P*_*i*-1_, *P*_*i*_) = 1, we have FDR(*i*) = *E*(*V*_*i*_). Furthermore

*E*(*V*_*i*_) = *P*(*V*_*i *_= 1)

since *V*_*i *_is a binary variable. Thus FDR(*i*) provides an unbiased estimation of *P*(*V*_*i *_= 1), the probability for gene *i *to be a false positive.

The raw local FDR estimate for gene *i *is:


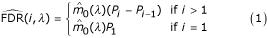


Assume that *H*_0_(*i*) and *H*_0_(*i *- 1) are true and *E*(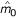) = *m*_0_. Therefore this estimate is unbiased with mean 1.

Using definition (1), it is direct to obtain:


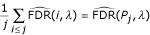


which equals the *q*-value of gene *j*. The *q*-value may thus be viewed as the mean of the raw local FDR of the genes with *p*-values lower than *P*_*j*_.

Under the hypothesis *H*_0_, it is known that the differences between successive ordered values of independent realizations of the uniform([0,1]) distribution have a Beta distribution with parameters 1 and *m*_0 _(see Johnson [9] Chap. 26). Therefore the variance of the raw local FDR estimate for non-differentially expressed genes when *m*_0 _is known is equal to 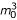/[(*m*_0 _+ 1)^2 ^(*m*_0 _+ 2)] ≈ 1, for *m*_0 _large enough.

The variance of estimates (1) under *H*_1 _is generally much smaller than under *H*_0 _(see Figures 1(a), 2(a) and 3(a) for an illustration). However, one may see on these Figures that 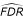
(*i*, λ) is a very variable estimator.

This fact is well known in point process literature, [8]. Moreover, the interval ]*P*_*i*-1_, *P*_*i*_[ is not symmetric. If we consider the neighborhood interval around *P*_*i *_defined by *t*_1 _= (*P*_*i*-1 _+ *P*_*i*_)/2, *t*_2 _= (*P*_*i*+1 _+ *P*_*i*_)/2 then we obtain another estimate of the local FDR:


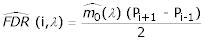


Note that (2) is a moving average of order 2 of (1). It is well known that estimates provided by moving average (or kernel estimators) are more stable, see [8].

This smoothing is generally not enough to obtain usable results and we can consider any kind of smoothing. We propose to estimate FDR(*i*) by


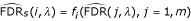


where *f*_*i *_is a smoothing function of the 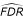
(*j*, λ) for *j *= 1, *m*, computed at position *P*_*i*_.

The smoothing method must be suited to the properties of the raw FDR:

• its variance is low for low *p*-values corresponding to highly differentially expressed genes

• its variance is very high for *p*-values corresponding to non differentially expressed genes

Therefore the window of smoothing should be short for low *p*-values and large for *p*-values corresponding high *p*-values. The lowess smoothing method has a fixed number of neighbor points. Therefore its window size depends of the density of points around the *p*-value under concern. The density of points is higher for low *p*-values which in turn implies a shorter window size, which is a good property. However the adaptation of the window size is not sufficient in some cases such as in the Apo-AI example. Moreover the smoothed FDR should be an increasing function of the *p*-values, a property which is not satisfied by the lowess smoothing. Therefore we prefer to use an *ad hoc *moving average smoothing using the following algorithm for computing 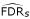
(*i*, λ): let 0 <*t*_1 _<*t*_2 _<*t*_3 _be three pre-definite thresholds and *m*_1 _<*m*_2 _<*m*_3 _<*m*_4 _four pre-definite integers.

• if max_*j*≤*i *_
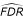
(*j*, λ) <*t*_1 _use a moving average of order *min*(2*i *- 1, *m*_1_)

• if *t*_1_ < max_*j*≤*i *_
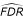
(*j*, λ) <*t*_2 _use a moving average of order *min*(2*i *- 1, *m*_2_)

• if *t*_2 _< max_*j*≤*i *_
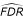
(*j*, λ) <*t*_3 _use a moving average of order *min*(2*i *- 1, *m*_3_).

• if max_*j*≤*i *_
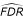
(*j*, λ) >*t*_3 _use a moving average of order *min*(2*i *- 1, *m*_4_).

We have obtained good empirical results on many data sets with *t*_1 _= 0.01, *t*_2 _= 0.05, *t*_3 _= 0.2, *m*_1 _= 3, *m*_2 _= 5, *m*_3 _= 15 and 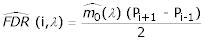 with the constraint that 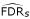
(*i*, λ) is not decreasing. This adaptative moving average method is quite empirical. This topic deserve some more work to build a well assessed smoothing method. This is one of our ongoing research project.

## Authors' contributions

Avner Bar-Hen, Jean-Jacques Daudin and Stephane Robin equally contributed to the statistical work and the redaction task. Julie Aubert coded the R-program and analyzed the three data sets.

## Figures and Tables

**Table 1:** *p*-value, *q*-value and local FDR estimates for three genes in Hedenfalk data.

gene	*p*-value	rank	*q*-value	raw local FDR	smoothed local FDR
MSH2	0.00005	8	0.013	0.013	0.010
PDCD5	0.00048	47	0.022	0.013	0.033
CTGF	0.0036	159	0.049	0.176	0.098

**Table 2:** Classification of m genes using threshold

	*H*_0 _accepted	*H*_0 _rejected	Total
*H*_0 _true	*U*(*t*)	*V*(*t*)	*m*_0_
*H*_0 _false	*T*(*t*)	*S*(*t*)	*m*_1_

Total	*W*(*t*)	*R*(*t*)	*m*
