# Correction: Determination of the differentially expressed genes in microarray experiments using local FDR

**DOI:** 10.1186/1471-2105-6-42

**Published:** 2005-03-03

**Authors:** J Aubert, A Bar-Hen, JJ Daudin, S Robin

**Affiliations:** 1 UM R INAPG/INRA/ENG REF 518, 16, rue C. Bernard, 75231 Paris Cedex 05, France

Following publication of this work [1], the authors became aware that Local FDR has been originally defined by Efron & al. (2001) in a mixture model framework [2].

## References

[B1] Aubert J, Bar-Hen A, Daudin JJ, Robin S (2004). Determination of the differentially expressed genes in microarray experiments using local FDR. BMC Bioinformatics.

[B2] Efron B, Tibshirani R, Storey J, Tusher V (2001). Empirical Bayes analysis of a microarray experiment. J Am Stat Assoc.

